# Health-Related Quality of Life Outcomes in Meningioma Patients Based upon Tumor Location and Treatment Modality: A Systematic Review and Meta-Analysis

**DOI:** 10.3390/cancers15194680

**Published:** 2023-09-22

**Authors:** Ali San, Raphia K. Rahman, Praveen Sanmugananthan, Michael D. Dubé, Nicholas Panico, Ogechukwu Ariwodo, Vidur Shah, Randy S. D’Amico

**Affiliations:** 1 College of Osteopathic Medicine, Kansas City University, Kansas City, MO 64106, USA; 2 Department of Neurological Surgery, Riverside University Health System, Moreno Valley, CA 92501, USA; 3 Northeast Ohio Medical University, Rootstown, OH 44272, USA; 4 Lake Erie College of Osteopathic Medicine, Erie, PA 16509, USA; 5 Philadelphia College of Osteopathic Medicine, Moultrie, GA 31768, USA; 6 Department of Neurological Surgery, Lenox Hill Hospital, Donald and Barbara Zucker School of Medicine at Hofstra/Northwell, New York, NY 11030, USA; rdamico8@northwell.edu

**Keywords:** meningioma, quality of life, 36-Item Short Form Health Survey, radiosurgery, skull base, craniotomy

## Abstract

**Simple Summary:**

We provide the first comprehensive systematic review and meta-analysis examining the impact of tumor location and treatment modalities on health-related quality of life (HRQoL) in patients with meningioma. We systematically reviewed HRQoL outcomes for non-skull base meningiomas (NSBMs), anterior, middle, and posterior fossa skull base meningiomas (SBMs) following neurosurgical and/or radiotherapeutic intervention. The literature was searched for studies that investigated HRQoL following surgical resection, radiosurgery, or radiotherapy and was analyzed to reveal HRQoL outcomes based on tumor location. With regard to therapeutic intervention, craniotomy was associated with significantly higher improvements in postoperative Karnofsky Performance Status (KPS) scores following treatment of anterior/middle skull base meningiomas (SBMs) in comparison to posterior SBMs. The authors further investigate factors that may impact HRQoL and the occurrence of postoperative neurological deficits. Due to a lack of consensus on the use of standardized quantitative tools to measure HRQoL, results are difficult to compare within the literature. We therefore provide recommendations on the utility of pertinent HRQoL instruments based upon skull base region and treatment modality.

**Abstract:**

Patients with meningiomas may have reduced health-related quality of life (HRQoL) due to postoperative neurological deficits, cognitive dysfunction, and psychosocial burden. Although advances in surgery and radiotherapy have improved progression-free survival rates, there is limited evidence regarding treatment outcomes on HRQoL. This review examines HRQoL outcomes based on tumor location and treatment modality. A systematic search in PubMed yielded 28 studies with 3167 patients. The mean age was 54.27 years and most patients were female (70.8%). Approximately 78% of meningiomas were located in the skull base (10.8% anterior, 23.3% middle, and 39.7% posterior fossae). Treatment modalities included craniotomy (73.6%), radiotherapy (11.4%), and endoscopic endonasal approach (EEA) (4.0%). The Karnofsky Performance Scale (KPS) was the most commonly utilized HRQoL instrument (27%). Preoperative KPS scores > 80 were associated with increased occurrence of postoperative neurological deficits. A significant difference was found between pre- and post-operative KPS scores for anterior/middle skull base meningiomas (SBMs) in comparison to posterior (SBMs) when treated with craniotomy. Post-craniotomy SF-36 scores were lower for posterior SBMs in comparison to those in the anterior and middle fossae. Risk factors for poor neurological outcomes include a high preoperative KPS score and patients with posterior SBMs may experience a greater burden in HRQoL.

## 1. Introduction

Meningiomas comprise 13–26% of all intracranial neoplasms [1,2]. Advances in radiation and surgical options for patients with symptomatic meningiomas have improved therapeutic outcomes and 5-year progression-free survival now approaches 90% [1]. However, many patients present with evidence of neurocognitive decline due to peritumoral edema and mass effect with impairments in executive functioning, working/verbal memory, information processing capacity, psychomotor speed, and self-perceived general health [3,4,5]. Following surgical resection, up to 40% of patients experience cognitive or emotional dysfunction with reports of these negative effects persisting even at five years postoperatively [6]. These negative effects in combination with or secondary to postoperative neurological deficits and their associated additional psychosocial burden can result in a decline in the health-related quality of life (HRQoL) of meningioma patients [5,6,7].

Historically, the Karnofsky Performance Scale (KPS) has been used to assess HRQoL in meningioma patients, with preoperative KPS scores predicting postoperative outcomes following resection [8,9,10,11]. Although a reliable tool to assess functional outcomes and physical quality of life, KPS is not a subjective tool and does not account for other factors of HRQoL including psychological and social well-being.

HRQoL is a multifactorial measure of a patient’s psychological, cognitive, and social functioning [5]. Validated instruments such as the Medical Outcomes Study 36-Item Short Form (SF-36) and the Sinonasal Outcome Test (SNOT-22) may capture components unaddressed by the use of KPS [12]. The SF-36 survey includes eight subscales that assess a patient’s physical activity, social activity, role limitations due to physical issues, mental health, role limitations due to emotional problems, pain, vitality, and general health [13]. The SNOT-22 is another patient-reported outcome measure (PROM), used primarily for anterior skull base intervention and is based on symptom severity, social impact, emotional impact, productivity, and influence on sleep [14].

The use of Core Outcome Sets (COS) outlines the essential minimum outcomes for measurement and reporting in clinical trials. While the creation of COS has played a crucial role in shaping research design, no such sets have been established within the realm of neuro-oncology [15]. As such, there is no current consensus on the use of a quantitative standardized tool to measure HRQoL in patients with meningioma, making it difficult to compare results within the literature due to heterogeneity [5]. Furthermore, there is a paucity of studies investigating differences in HRQoL outcomes based on tumor location [16]. We systematically reviewed HRQoL and neurocognitive outcomes for non-skull base meningiomas (NSBMs), and anterior, middle, and posterior fossa skull base meningiomas (SBMs) following neurosurgical and/or radiotherapeutic intervention. 

## 2. Methodology

**Figure 1:**
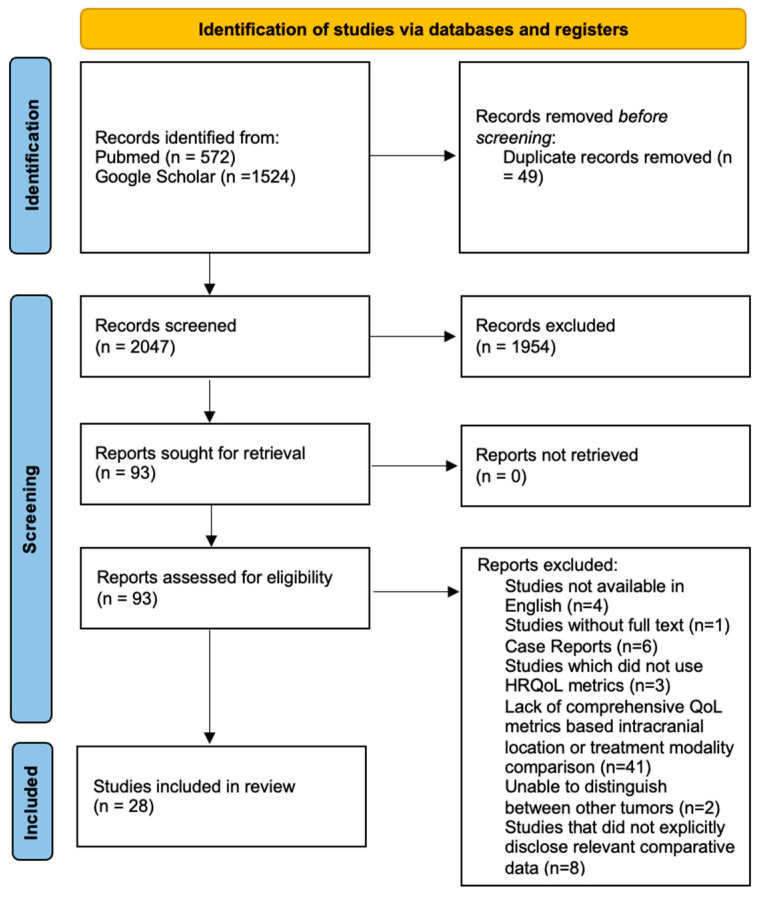
PRISMA flowchart of systematic search strategy for Health-Related Quality of Life Outcomes in Meningioma Patients Based Upon Tumor Location and Treatment Modality.

A literature search was conducted using PubMed and Google Scholar databases) to identify relevant articles published between January 1984 and December 2022 using PRISMA (Preferred Reporting Items for Systematic Reviews and Meta-Analyses) guidelines (Figure 1). These references were reviewed by three independent reviewers. The search criteria included the following terms in the PubMed database:

1. PubMed: ((Quality of life)) AND meningioma resection: 144 results (19 included).
2. PubMed: ((Quality of life)) AND meningioma therapy: 288 results (3 included).
3. PubMed: (“meningioma*”[Title/Abstract] AND (“postoperative KPS”[All Fields] OR “postoperative Karnofsky performance”[All Fields])): 43 results (5 included).
4. PubMed: (“meningioma*”[Title/Abstract] AND (“postoperative SF-36”[All Fields]) OR “postoperative 36-Item Short Form Survey”[All Fields])): 3 results (0 included).
5. PubMed: (“meningioma*”[Title/Abstract] AND (“endoscopic endonasal approach”[All Fields]) OR “postoperative SNOT-22”[All Fields])): 22 results (0 included).
6. PubMed: (“meningioma*”[Title/Abstract] AND (“instrument”[All Fields] OR “postoperative EORTC QLQ-BN20”[All Fields] OR “postoperative SNOT-22”[All Fields] OR “postoperative ASBQ”[All Fields] OR “postoperative EORTC QLQ-C30”[All Fields] OR “postoperative EQ-5D”[All Fields] OR “postoperative IHD”[All Fields] OR “postoperative NHP”[All Fields] OR “postoperative FACT-BR”[All Fields] OR “postoperative HADS”[All Fields] OR “cognitive function”[All Fields])): 72 results (6 duplicates, 0 included).
7. Google Scholar: meningioma AND quality of life assessment OR quality of life questionnaire OR KPS or SF-36: 217 results (1 included).
8. Google Scholar: meningioma AND endoscopic endonasal approach OR postoperative SNOT-22: 406 results (3 duplicates, 0 included).
9. Google Scholar: meningioma AND postoperative SF-36 OR postoperative 36-Item Short Form Survey: 212 results (8 duplicates, 0 included).
10. Google Scholar: ((health-related quality of life)) AND meningioma craniotomy: (16 duplicates, 0 included).

We assessed article quality, study type, and patient outcomes. Inclusion criteria specified studies that utilized surgical resection, radiosurgery, or radiotherapy for the treatment of intracranial meningiomas and reported HRQoL metrics, as measured by quality-of-life assessment instruments. Furthermore, studies that included adult and or pediatric populations were included. 

Exclusion criteria involved studies that did not explicitly disclose relevant qualitative or comparative data for subjects (*n* = 8), incomprehensive studies that did not report QOL metrics based upon intracranial location or treatment modality (*n* = 41), studies in which patient data were unable to be distinguished from other brain lesions including but not limited to gliomas (*n* = 2), studies that solely examined cognitive dysfunction without utilizing an HRQoL instrument (*n* = 3), case reports (*n* = 6), those unavailable in English (*n* = 4) and those without full-text availability (*n* = 1). Relevant studies were included from article references when applicable (*n* = 6). This resulted in 65 references being discarded, leaving 28 studies (Figure 1). 

Information extracted included age, sex, presenting symptoms, meningioma grade and location, nidus size, extent of resection (EOR), treatment modality, QoL metrics, postoperative neurologic deficits, recurrence, mortality, and median follow-up time. For skull base meningiomas, tumor locations were categorized based on Al-Mefty’s definition of SBMs [17]. Mortality was evaluated based on direct or indirect results of treatment, and studies that did not specify cause of death were excluded from data analysis. The type and number of instruments used to assess HRQoL were extracted and analyzed. In instances where studies used multiple metrics to assess HRQoL, each metric was reported. Questionnaire scores were extracted from each study and compiled to determine effects of treatment on HRQoL. For studies that reported 36-Item Short Form Health Survey (SF-36) results, patients were separated based on tumor location to determine its impact on quality of life; weighted means of each SF-36 component were calculated based on tumor location [12,16,18,19,20]. Studies that reported pre- and post-operative Karnofsky Performance Scale (KPS) scores were additionally compiled and analyzed [20,21,22,23,24,25,26]. Even though some studies utilized more than one metric, only studies that utilized SF-36 and KPS metrics were analyzed and compiled into graphs as these metrics were most commonly used and had sufficient data that met inclusion criteria. Studies that met inclusion criteria but contained different QoL metrics were not compiled in a table or graph but were included in the overall demographics of the paper. Lastly, a risk of bias assessment using ROBINS-I was performed to determine the risk of bias of each study (Table A1) [27]. 

A meta-analysis of proportions was conducted to determine the impact of pre-operative KPS score on post-operative neurological deficits. Patients were divided into the following groups: those who had a pre-operative KPS score of >80 and those who had a preoperative KPS score of ≤80. The data were transformed using the arcsine method to determine variance [28]. Heterogeneity was determined using the DerSimonian-Laird random effects model method. The pooled data were then back-transformed and heterogeneity was assessed using the Cochran Q and I^2^ tests. A meta-regression was performed on continuous variables to determine the cause of heterogeneity using the following moderators: WHO Grade, EOR, location, sex, nidus size, and treatment. Furthermore, a subgroup analysis was performed on the following categorical variables: treatment, study type, tumor location, and study population. A funnel plot and Egger’s regression test were applied to determine publication bias [29]. Finally, standard mean difference was used to compare pre- and post-KPS mean scores in patients treated with craniotomy in the posterior and anterior/middle fossa. If a study failed to provide the standard deviation for mean KPS scores, it was excluded from the analysis. This situation applied to studies utilizing EEA, where the standard mean difference or individual patient data points could not be computed and consequently was not incorporated into our meta-analysis. A value of *p* < 0.05 was deemed as significant. All statistical analyses were performed using meta and metafor packages in RStudio version 4.2.2 and R. This study has not been registered.

## 3. Results

### 3.1. Overview 

Using the PubMed and Google Scholar electronic databases, 2047 articles were screened from the literature, of which 28 articles met inclusion criteria. A summary of these studies is available in Table A2. Patient demographics are presented in Table 1. The mean age was 54.27 years (range 3–87 years) with a higher prevalence of females (70.8%). The median follow-up was 51.58 months. When reported, the most common symptoms at presentation were cranial nerve palsies (42.5%) and visual disturbances (13.3%). Less frequently reported symptoms included headaches (9.7%) and seizures (4.7%). Cognitive deficits were present in 0.7% of the cohort and 2.8% and 0.8% of patients reported motor and sensory disturbances, respectively. Notably, 10 studies did not report information on presenting symptoms [22,23,30,31,32,33,34,35,36,37]. 

World Health Organization (WHO) Grade was reported in the majority of studies. Of these, 98.7% were classified as I-II with a mean tumor size of 39.1 mm. Approximately 78% of meningiomas arose from the skull base, including the anterior (10.8%), middle (23.3%), and posterior (39.7%) fossae. The most common treatment method was craniotomy (73.6%) followed by first-line radiotherapy (11.4%). Combination therapy with surgical resection and adjuvant radiotherapy was reported in 9.2% of patients. Less commonly used therapies included EEA for tumor resection (4.0%). The extent of resection (EOR) was reported as gross total resection (GTR) or Simpson Grade I-III in 62% of patients. Of the studies that reported intraoperative complications, the total incidence of intraoperative complications was 21.8% and the most common complication was CSF leak and infection (21.1%). Transient postoperative complications (6.0%) included cranial nerve (30.7%) and motor/language deficits (14.8%). On follow-up, 55.2% of patients had new-onset or permanent deficits including cranial nerve (64.3%), motor/language deficits (6.9%), or hemiparesis (7.6%). Mortality as a result of intervention or from postoperative complications occurred in 2.4% of the total patient population. Tumor recurrence was reported in a total of 326 patients (10.3%). 

### 3.2. QoL Metrics

A variety of instruments were used to measure HRQoL, with the 36-Item Short Form Health Survey (SF-36) (20%) and Karnofsky Performance Scale (KPS) (27%) being the most frequently utilized (Table 2). Instruments categorized as “Other” (20%) were heterogeneous and lacked quantifiable data, and thus were excluded from the meta-analysis [18,30,33,36,37]. Additionally, 35.7% of studies utilized more than one instrument to measure QoL [16,18,20,32,35,36,37,38,39,40]. 

Five studies (*n* = 356 patients) reported pre- and post-KPS scores [20,21,24,41,42]. Only studies that reported average KPS scores and standard deviations were included in this analysis. Data were divided into two distinct groups consisting of patients who underwent craniotomy for anterior/middle SBMs and those who underwent craniotomy for posterior SBMs. Standard mean difference analysis revealed a significant difference between pre- and post-KPS scores following craniotomy based on intracranial location. Patients with meningiomas located in the anterior/middle fossa had significantly higher postoperative KPS scores (−1.16; 95% CI, [−1.99–0.33; *p* = 0.0061) (Q = 1.65, *p* = 0.1985, I^2^ = 39.51%) [24,41], whereas those in the posterior fossa did not show significant improvement in KPS postoperatively (−0.13; 95% CI, [−0.63–0.38], *p* = 0.6145)(Q = 17.87, *p* = 0.0005, I^2^ = 83.21) [21,24,42]. Statistical tests were not performed for the EEA group as there was only one study that met the inclusion criteria. However, it is also important to note that due to the low number of studies included in this analysis, more long-term studies investigating HRQoL and functional outcomes are required in the literature.

A total of four studies (*n* = 285 patients) reported post-craniotomy SF-36 scores by location, not including physical component scores (PCS) and mental health score (MCS) scores (Table 3) [12,16,19,20]. It is important to note that preoperative SF-36 scores were unreported and thus unavailable for analysis. Three studies investigated QoL using SF-36 for posterior fossa meningiomas (*n* = 110 patients), one study (*n* = 62 patients) analyzed both anterior and middle fossae, while one study analyzed NSBMs (*n* = 84 patients) (Table A2) [12,16,19,20]. Due to the low power of studies in the anterior/middle and NSB groups (*n* = 1), statistical analysis was not performed between the three locations [16]. The HRQoL questionnaire was obtained at a mean of 46.5 months postoperatively (range: 5–108 months). In comparison to anterior/middle and NSBMs, Table 3 shows a trend of lower scores in all components of the SF-36 assessment for posterior fossa meningiomas, with noticeable decreases in the components of physical functioning, role limitation due to physical and emotional health, energy, social function, and general health. Patients with meningiomas of the anterior/middle fossa display a trend of higher scores for the component of general health in comparison to other cranial locations. Those with NSBMs had a trend of lower scores in components including physical functioning, role limitation due to physical and emotional health, energy, and social function categories.

**Figure 2:**
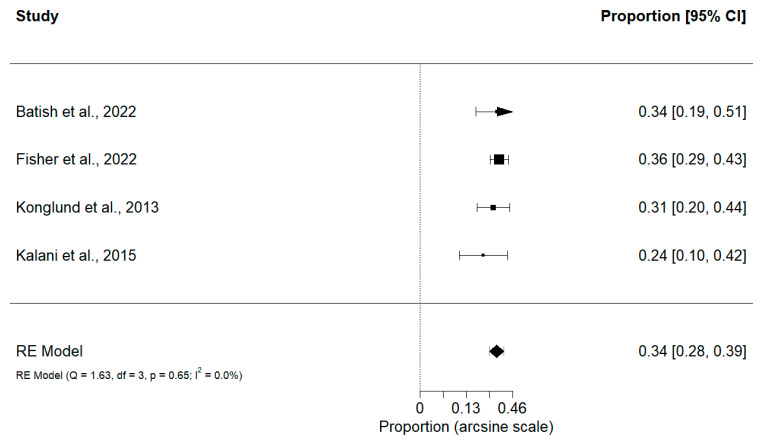
Proportion of patients that experience postoperative neurological deficits based on KPS > 80 [16,20,24,32].

**Figure 3:**
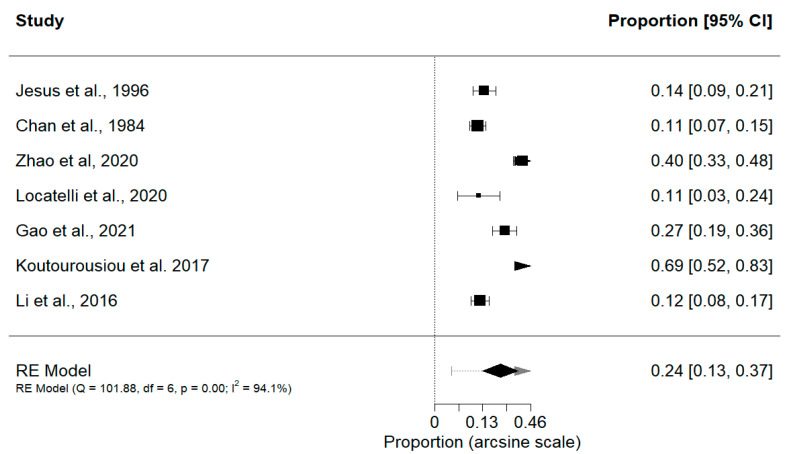
Proportion of patients that experienced postoperative neurological deficits based on KPS ≤ 80 [21,26,31,40,41,42,44].

Preoperative KPS scores > 80 had a slightly greater proportion of postoperative deficits (0.34; 95% CI, 0.28–0.39; *p* < 0.0001) (Q = 1.63, *p* = 0.65, I^2^ = 0.00%) compared to scores ≤ 80 (0.24; 95% CI, 0.13–0.37; *p* < 0.0001) (Q = 101.9, *p* < 0.0001, I^2^ = 94.11%) (Figure 2 and Figure 3). It is important to note that there was significant heterogeneity in the group with lower preoperative KPS scores; therefore, a meta-regression was performed to determine the impact of specific moderators on heterogeneity such as WHO Grade, EOR, sex, and nidus size. Furthermore, a subgroup analysis was performed on the following categorical variables: treatment, study type, tumor location, and study population. Analysis for meta-regression revealed that none of the continuous variables significantly contributed to the heterogeneity observed in our study, which may be due to the low power in this study. López-López et al. [43] suggest that the number of studies highly contributes to the accuracy of the model, where at least 20 studies are recommended. As the current study does not meet this threshold, it is possible that the true effect of each moderator on heterogeneity may not accurately be portrayed. Conversely, subgroup analysis of categorical variables revealed a significant difference in treatment type (*p* = 0.0028) in the >80 KPS group and a significant difference in the treatment modality (*p* < 0.0001) and location (*p* = 0.0241) subgroups in the <80 KPS group.

## 4. Discussion

To the authors’ knowledge, this is the first systematic review and meta-analysis to investigate QoL based on meningioma location and treatment type. Despite different therapies, QoL outcomes remain mostly unchanged after therapy in examined studies [8,12,20,23,31,44,45,46]. However, craniotomies may provide a significant impact on QoL in patients with anterior/middle SBMs in comparison to posterior SMBs [24,25]. Additional prospective studies are needed to conclude whether EEA may provide the same benefits in this population. Tumor location and treatment type likely play key roles in predicting QoL outcomes. Studies have attempted to utilize a variety of HRQoL metrics to better predict and prognosticate QoL in meningioma patients. While the creation of “core outcome sets” (COS) has played a crucial role in shaping research design for clinical trial effectiveness, no such sets have been established within the realm of neuro-oncology. However, no standardized measure of HRQoL has been consistently utilized making the available data difficult to interpret. 

Tumor location and other factors such as preoperative KPS scores may influence postoperative HRQoL. Our review found that patients with higher preoperative KPS scores experienced more neurologic deficits following resection, thereby influencing QoL outcomes. This finding may reflect the fact that patients with KPS scores > 80 have normal activity, with minor to some signs and symptoms of disease, and, therefore, are at greater risk of losing functional status postoperatively in comparison to those with a lower functional status at baseline. Furthermore, tumor location may determine the extent of burden on HRQoL and the occurrence of neurological deficits. For example, tumors of the posterior fossa have been demonstrated to be associated with decreased HrQoL and increased morbidity [16,47]. This is supported by our analysis, which demonstrates no significant difference in the standard mean difference between pre- and post-KPS scores following craniotomy for resection of posterior fossa meningiomas. Early intervention for posterior fossa SBMs may likely play a role in limiting the spread and size of tumors in an already difficult location. However, it is important to note that our analysis revealed large heterogeneity in the <80 KPS group, thereby limiting the ability to determine the true relationship between KPS score and postoperative deficits. Furthermore, subgroup analysis demonstrated that treatment type along with tumor location may influence the occurrence of postoperative neurological deficits. Additionally, the number of symptoms at diagnosis has been negatively correlated with other HRQoL assessments including the Functional Assessment of Cancer Therapy (FACT) and thus may be used as a QoL predictor as symptoms may persist despite therapy [48]. Further evidence is required to understand factors that may impact QoL following treatment in this population. It is important to note that the use of various QoL metrics for tumors in the same intracranial location limits the comparison of QoL data. Furthermore, to uniformly assess QoL metrics, the utility of HRQoL instruments should reflect both tumor location and treatment modality (Table 4). 

### 4.1. Skull Base vs. Non-Skull Base Meningiomas 

Skull base meningiomas (SBMs) require complex microsurgical techniques due to the tumor’s relationship with critical neurovascular structures, leading to an increased risk of neurologic deficits including cranial nerve palsies and brainstem dysfunction after gross total resection (GTR). Preoperatively, patients with SBMs have lower HRQoL scores and more neurological deficits than those with non-skull base meningiomas (NSBM) [9,49]. In our analysis, patients with NSBMs had a higher average score for mental health in comparison to anterior/middle and posterior SBMs postoperatively (Table 3). This may reflect the fact that patients with SBMs have a higher incidence of preoperative neurologic deficits than those with NSBMs, which may predispose them to a significant mental health burden. However, average SF-36 scores for the components of role limitation due to emotional health, energy, social function, pain, general health, and physical/mental components were similar between those with NSBMs and SBMs. 

Using the SF-36 assessment, Fisher et al. [16] found no significant difference in HRQoL between patients with SBM (*n* = 89) and those with convexity meningiomas (*n* = 84) following resection. In contrast, Miao et al. [34] found significant differences in outcomes based on tumor location (*p* < 0.008) using the World Health Organization Quality of Life-100 scale (WHOQOL-100). Patients with NSBMs in the falcine, convexity, and parasagittal regions had significantly higher QoL than those with SBMs in the clivus, olfactory groove, and sphenoid ridge postoperatively. Furthermore, studies show that SBM patients have significantly higher rates of retreatment and neurological deficits (i.e., increased motor/visual deficits and cognitive complaints) than those with convexity meningiomas, which may impact QoL [50]. However, due to conflicting findings among studies, it is undetermined whether these factors decrease QoL [16,49,51]. We hypothesize that the differences in QoL in these studies and our analysis may be due to the heterogeneity of metrics utilized during patient assessment. Standardization of HRQoL assessments may further augment the ability to prognosticate QoL outcomes in these patient populations. Further studies are required to determine the impact of tumor location on HRQoL outcomes, considering confounders such as tumor size and EOR.

### 4.2. Anterior/Middle Skull Base Meningiomas

Compared to other intracranial locations, anterior and middle fossa SBMs show higher SF-36 scores for physical functioning, role limitation due to physical health, role limitation due to emotional health, energy, social function, general health, and mental component summary (Table 3). Our results corroborate Lauridsen et al.’s. [48], where the authors showed that patients with subfrontal meningiomas experienced lower anxiety and depression than patients with other intracranial meningiomas as evaluated by the Hospital and Anxiety Scale (HADS). This is further supported by our analysis, which showed a significant difference between means of pre- and postoperative KPS scores for meningiomas in the anterior/middle fossa. Furthermore, anterior/middle meningiomas provide unique access for the endoscopic endonasal approach [24,25]. Seven studies reported QoL outcomes for anterior SBMs after surgical resection, with three using an endoscopic approach [16,24,25,30,34,38,39]. Two studies using EEA found a significant difference between preoperative and long-term postoperative KPS scores [41,52]. With careful monitoring of sinonasal outcomes, EEA may serve as an alternative method of resection. Using SNOT-22 and the Anterior Skull Base Questionnaire (ASBQ), Kirszbaum et al. [39] investigated sinonasal-specific and overall QoL respectively after EEA in 50 patients. With initially worst sinonasal outcomes postoperatively, outcomes gradually returned to preoperative values at 6-week follow-up. Larger tumors and visual dysfunction negatively impacted QoL, but improvements in vision and lower preoperative ASBQ scores predicted QoL improvements at 6 months. Olfaction and taste worsened following surgery but did not correlate with overall QoL. 

Similarly, Jones et al. [38] investigated long-term QoL after EEA in 34 patients using univariate analysis of factors including age, EOR, and previous treatments. Age > 55 years was significantly associated with lower ASBQ scores, indicating that age is an important factor in QoL. In this age group, radiosurgery or observation may be considered to preserve QoL. There was a significant deterioration in sinonasal-specific QOL after EEA, with no significant differences in pre- and postoperative ASBQ domain scores. Further studies are required to determine whether long-term sinonasal outcomes are preserved following EAA.

In patients with anterior SBMs, lesions specifically of the ventromedial prefrontal cortex (vmPFC) may lead to the development of acquired personality disturbances (APD) contributing to impaired adaptive functioning including independence, occupational functioning, interpersonal relationships, and emotional equilibrium [30]. Patients with vmPFC lesions had significantly worse adaptive functioning and higher rates of APDs for apathy, blunted affect, irritability, and poor judgment following resection. Furthermore, patients with vmPFC lesions had significantly increased depression scores on QoL assessments. It may be beneficial to screen for APDs using a personality test such as the Iowa Scales of Personality Change (ISPC) in the setting of a multidisciplinary team including psychiatry and neurology in this population.

Furthermore, meningiomas of the vmPFC may impact executive functioning, and thus select neuropsychological battery tests including the Central Nervous System Vital Signs tests may be utilized as an adjunct in evaluating the extent of functional impairment [53]. In patients who underwent craniotomy for olfactory groove meningiomas, Constanthin et al. [54] reported a significant reduction in attention, flexibility, and language immediately after surgery, with continued impairment in cognitive outcomes upon one-year follow-up. However, categories assessing disinhibition, updating, memory, and orientation, which immediately decreased after surgery, returned to baseline levels upon one-year follow-up [54]. The use of EEA for anterior meningiomas may translate into improvement in neuropsychological battery testing scores, as well as a decrease in the development of APDs. However, further research is required to elucidate whether KPS scores are predictive of neurocognitive and psychological outcomes following treatment with EEA versus craniotomy. 

The European Association of Neurological Surgery (EANS) created a task force in 2019 to discuss the use of EEA versus transcranial craniotomy in the management of tuberculum sellae meningiomas (TSBs). The committee recommended the use of a transcranial approach over EEA for TBS due to EEA’s limited surgical freedom and inability to control vascular injury [55]. It is important to note that certain advantages of EEA include early decompression of the optic canal, devascularization of tumors, avoidance of brain retraction, and decreased incidence of new neurological deficits [55,56]. However, it is unclear whether these benefits may outweigh its disadvantages. Additionally, few studies demonstrate EEA to be associated with improved visual dysfunction, which may in turn positively correlate with QoL [39,57,58]. 

Common intraoperative complications associated with EEA for TBS resection include cerebrospinal fluid leak and worsening of olfaction, which is less commonly observed with other midline anterior SBMs [39,55]. Jones et al. [38] found that reductions in olfaction and taste did not correlate with overall QoL following surgery. However, due to a limited number of studies, it is difficult to conclude which factors are significant predictors of HRQoL following EEA. Therefore, it is recommended to consider tumor size, location and accessibility, the incidence of postoperative complications including onset of new neurological deficits, and patient expectations when selecting a surgical technique for tumor resection. Further prospective studies are required to elucidate HRQoL outcomes for EEA versus other surgical techniques in the resection of anterior SBMs.

One study investigated QoL in middle fossa SBMs and found no significant difference in KPS scores for meningiomas involving the cavernous sinus at 3- and 12-month follow-up postoperatively [31]. However, results are difficult to interpret given the limitations of KPS as a metric of HRQoL. 

### 4.3. Posterior Skull Base Meningiomas 

Definitive surgical management of petroclival meningiomas (PCMs) is challenging due to their proximity to vital neurovascular structures and a high associated risk of morbidity and mortality [59]. Postoperative neurological deficits are common, including cranial nerve palsies, trigeminal neuropathy, hearing loss, and impaired vision, leading to a decline in performance status [60]. Compared to anterior/middle fossa SBMs, PCM patients displayed a trend of lower QoL scores, particularly in physical functioning, role limitation, energy, social function, general health, mental health, and physical component summary (Table 3). Furthermore, analysis of pre- and postoperative KPS means did not find a significant difference in posterior meningioma studies. Our findings corroborate prior findings in the literature. In a study by Fisher et al., patients with anterior/middle SBMs had significantly better QoL than those with posterior SBMs as measured by SF-36, specifically in role limitations due to physical functioning. However, no significant difference was found in neurocognitive functioning between anterior/middle and posterior SBMs, which may be attributed to cerebral cortex functioning being diffusely distributed over the brain surface [16]. The authors recommend the use of EEA in select petroclival meningiomas as it may significantly increase HRQoL [26]. 

The relationship between the tumor and cranial nerves and the potential for cranial nerve deficits may influence patients’ perceived quality of life following resection, with deterioration in psychosocial functioning [19]. In a series of transpetrosally operated PCMs (*n* = 29), cranial nerve dysfunctions on follow-up included trigeminal symptoms (31%), moderate to moderately severe facial nerve dysfunction on House–Brackmann Grade (24%), little to no hearing (27%), and swallowing difficulties (17%). Patients with swallowing difficulties due to tumor invading the petrous bone and jugular foramen (affecting CN IX, X, and XI) reported low physical and mental health scores, while those with postoperative trigeminal nerve neuropathy had low physical health and social adjustment scores. In a study of those undergoing resection for PCMs (*n* = 46) and lateral posterior pyramid meningiomas (LPPM) (*n* = 32), postoperative SF-36 scores were significantly lower in comparison to those in a reference population in the domains of physical functioning, role-physical, general health, vitality, and social functioning [12]. This was attributed to the new onset of postoperative deficits including hemiparesis, swallowing difficulties, and hypoacusis. Intraoperative electrophysiological monitoring and adjuvant therapies such as radiation and systemic therapies may improve HRQoL outcomes in this population [20,61,62]. As a result, NTR may lead to better QOL outcomes than GTR for PCM patients. 

### 4.4. Therapeutic Strategies

Limited evidence exists on the long-term effects of radiotherapy/radiosurgery on HRQoL [46,63]. When comparing QoL in posterior SBMs, patients who underwent near total/subtotal excision with adjuvant adjuvant Gamma Knife Radiosurgery (GKRS) had better QoL scores in the general health domain than those who received GTR or NTR alone [20]. A paradigm of safe, near-total resection of SBM with adjuvant radiosurgery may maximize QoL metrics in this patient population.

Fractionated stereotactic radiotherapy or intensity-modulated radiotherapy improved HRQoL in 37.5% of patients with a reduction observed in 11% [64]. However, HRQoL was lower after radiotherapy in comparison to the normal population and meningioma patients treated by surgery alone, with decreased functioning and increased symptoms including fatigue and pain. In patients that received stereotactic photon radiotherapy, mean SF-36 values declined after therapy and normalized towards initial values at the 12-month follow-up [46]. Gender, age, and tumor-related symptoms were not found to significantly affect physical and mental component scale scores. Due to limited data, further studies are required to determine the effects on QoL outcomes based on tumor location when using radiotherapy. 

A few studies have investigated the effects of radiotherapy on cognitive outcomes—though, without the use of standardized tools [50,53,65,66]. Cognitive function was found to be worse in patients treated with radiotherapy compared to those who underwent surgery, particularly in verbal memory [8]. A transient decline in memory function was also observed after the first fraction of fractionated SRS, but no cognitive deterioration was detected on further follow-up [50,65,66]. Patients who underwent resection plus radiotherapy reported lower functioning, communication deficits, and a significant decline in verbal memory [15,33]. Patients may benefit from long-term monitoring of cognitive function with the use of adjuvant radiotherapy.

Our analysis demonstrates no significant improvement in postoperative KPS scores following treatment of posterior SBMs with craniotomy. This may be due to tumor location, which in turn affects the therapeutic strategy of choice. Meningiomas of the posterior fossa are associated with worse QoL outcomes [16], and such patients undergo invasive craniotomy with a greater burden of postoperative complications, thereby potentially confounding results. Therefore, when evaluating QoL outcomes in the clinical setting, the impact of tumor location and the chosen therapeutic strategy should be considered. However, it is important to note that due to the limited number of studies and lack of standardized QoL metrics, it is difficult to compare the efficacies of therapeutic approaches based on QoL outcomes. Further studies are required to investigate differences between therapeutic strategies and their impact on HRQoL, specifically in the anterior/middle cranial fossae.

### 4.5. Limitations

The heterogeneity in HRQoL metrics among the 28 studies reviewed contributed to the limitations of the present study. We analyzed scores from only 22 studies and were limited in comparing HRQoL scores based on tumor location, preoperative KPS scores, and other factors. In particular, preoperative SF-36 scores were unreported in all craniotomy studies and thus could not be compared to postoperative values. Furthermore, our analysis of KPS showed high heterogeneity due to uncontrolled confounders in the studies. In addition, subgroup analysis demonstrated that treatment type in the >80 KPS group, as well as treatment type and meningioma location in the <80 KPS group, impacted results pertaining to postoperative neurological deficits. Furthermore, there were limited studies included in the comparison of QoL outcomes following EEA due to the lack of standard deviation reporting of mean KPS scores. Differences in QoL outcomes between therapeutic strategies were unable to be investigated due to studies utilizing different HRQoL instruments (e.g., SNOT-22 vs. SF-36 for EEA vs. craniotomy, respectively), with minimal studies investigating differences in QoL metrics based upon therapeutic strategies. We were also unable to compare QoL outcomes in radiosurgery/radiotherapy studies in our analysis due to the limited number of quantifiable HRQoL scores reported. Future studies should investigate for potential confounding variables on QoL outcomes based on therapeutic strategy. Additionally, there is limited evidence regarding the long-term effects of treatment type on HRQoL outcomes in SBM patients, particularly those with NSBMs or those treated with radiotherapy/radiosurgery. More studies are required to determine long-term HRQoL and neurocognitive outcomes of different treatment approaches.

## 5. Conclusions

Patients undergoing treatment for meningioma may experience deterioration in HRQoL due to cognitive or emotional dysfunction, psychosocial burden, and postoperative neurological deficits. Posterior fossa SBMs may have a greater impact on HRQoL compared to other types of SBMs and NSBMs, as demonstrated by lower postoperative KPS and SF-36 scores. Radiotherapy’s effect on memory function should be evaluated, and alternative adjuvant therapies such as GKRS or EEA may be considered for posterior fossa SBMs to improve QoL outcomes. Craniotomy for anterior/middle SBMs demonstrates higher postoperative KPS scores in comparison to posterior SBMs; however, careful monitoring of subjective QoL and neurocognitive assessments are recommended by a multidisciplinary team. Risk factors for poor neurologic outcomes may include a higher preoperative KPS score; however, this may be confounded by treatment type and meningioma location. HRQoL instruments should be selected based on tumor location and treatment modality. Further research is needed to determine HRQoL outcomes in EEA and long-term outcomes of radiotherapy/radiosurgery. 

## Figures and Tables

**Table 1:** Patient Demographics.

**Total Number of Patients**	**3167**
**Mean Age ^a^**	54.27 ± 6.33
**Male:Female (*n*)**	926:2241
**WHO GRADE ^b^**	
** I–II** ** III**	2099 27
**Average Nidus Size (mm) ^c^**	39.10 ± 7.96
**Presenting Symptoms (%) ^d^**	
** Headache** ** CN Palsy** ** Seizure** ** Visual Disturbances** ** Motor Disturbances** ** Sensory Disturbances** ** Exophthalmia** ** Cerebellar Deficits** ** Nausea/Vomiting** ** Memory Disturbances** ** Cognitive Deficits** ** Ataxia** ** Asymptomatic** ** Other**	357 (9.72%) 1560 (42.5%) 174 (4.7%) 490 (13.3%) 104 (2.8%) 31 (0.8%) 113 (3.1%) 46 (1.3%) 33 (0.9%) 39 (1.1%) 25 (0.7%) 185 (5.0%) 75 (2.0%) 439 (12.0%)
**Treatment**	
** Craniotomy** ** Craniotomy + RT** ** RT** ** Endoscopic** ** Crani + Endo** ** Craniotomy + Radiosurgery**	2102 265 327 114 9 48
**Location**	
** SB** ** Anterior** ** Middle** ** Posterior** ** NSB**	2288 320 688 1173 664
**EOR ^e^**	
** GTR** ** Partial**	1582 986
**Average Median Follow-up (months) ^f^**	51.58 ± 43.8
**Surgical Complications (%) ^g^** ** CSF leak** ** Hydrocephalus** ** Infection** ** Hematoma** ** Hemorrhage** **Other**	399 84 (21.1%) 83 (20.8%) 84 (21.1%) 23 (5.8%) 12 (3.0%) 113 (28.3%)
**Postop deficits ^h^**	
** Transient** ** CN Deficit** ** Motor/Language Deficit** ** Neurologic Deficit** ** Other** ** Permanent** ** CN Deficit** ** Motor/Language Deficit** ** Neurologic Deficit** ** Eye Motility Deficit** ** Hemiparesis** ** Other** **Other/Unspecified**	189 58 (30.7%) 28 (14.8%) 26 (13.8%) 77 (40.7%) 462 297 (64.3%) 32 (6.9%) 13 (2.8%) 23 (5.0%) 35 (7.6%) 62 (13.4%) 185
**Mortality ^i^**	77
**Recurrence ^j^**	326

^a^ Four studies did not contain information on mean age (Jesus et al., 1996; Konglund et al., 2012; Neil-Dwyer et al., 2000; Timmer et al., 2019). ^b^ Nine studies did not contain information on tumor grade (Batish et al., 2022; Chan et al., 1984; Castle-Kirszbaum et al., 2022; Jones et al., 2016; Mathiesen, et al., 2007; Miao et al., 2009; Mohsenipour et al., 2001; Neil-Dwyer et al., 2000; Pintea et al., 2018). ^c^ Fourteen studies contained information on nidus size (Barrash et al., 2019; Batish et al., 2022; Fisher et al., 2022; Jones et al., 2016; Kalasauskas et al., 2021; Karsy et al., 2019; Mathiesen, Et al., 2007; Miao et al., 2009; Mohsenipour et al., 2001; Pintea et al., 2018; Zhao et al., 2020; Li et al., 2016; Gao et al., 2021; Koutourousiou et al., 2017). ^d^ Ten studies did not contain information on presenting symptoms (Barrash et al., 2020; Jesus et al., 1996; Konglund et al., 2012; Krupp et al., 2008; Miao et al., 2009; Mohsenipour et al., 2001; Pintea et al., 2018; Tariciotti et al., 2022; Timmer et al., 2019; Wirsching et al., 2020). ^e^ Three studies did not contain information on the extent of resection (Krupp et al., 2008; Mohsenipour et al., 2001; Neil-Dwyer et al., 2000). ^f^ Eight studies did not contain information on median follow-up (Kalasauskas et al., 2021; Konglund et al., 2012; Miao et al., 2009; Mohsenipour et al., 2001; Neil-Dwyer et al., 2000; Locatelli et al., 2020; Kalani et al., 2015; Qiao et al., 2019). ^g^ Eight studies did not contain information on surgical complications (Combs et al., 2013; Henzel et al., 2013; Kalasauskas et al., 2021; Miao et al., 2009; Mohsenipour et al., 2001; Tariciotti et al., 2022; Timmer et al., 2019; Wirsching et al., 2020). ^h^ Eight studies did not contain information on postoperative complications (Combs et al., 2013; Henzel et al., 2013; Kalasauskas et al., 2021; Miao et al., 2009; Mohsenipour et al., 2001; Tariciotti et al., 2022; Timmer et al., 2019; Wirsching et al., 2020). ^i^ Nine studies did not contain information on mortality (Combs et al., 2013; Fisher et al., 2022; Miao et al., 2009; Mohsenipour et al., 2001; Pintea et al., 2018; Timmer et al., 2019; Wirsching et al., 2020; Locatelli et al., 2020; Qiao et al., 2019). ^j^ Seven studies did not contain information on tumor recurrence (Combs et al., 2013; Henzel et al., 2013; Konglund et al., 2012; Krupp et al., 2008; Neil-Dwyer et al., 2000; Tariciotti et al., 2022; Timmer et al., 2019).

**Table 2:** Number of times QOL instruments were utilized by 28 studies to quantitatively measure health-related quality of life.

QOL Metric Used	Number of Times Utilized
**Institution Specific**	3
**KPS**	12
**SF-36**	10
**EORTC QLQ-BN20**	1
**SNOT-22**	2
**ASBQ**	2
**EORTC QLQ-C30**	2
**EQ-5D**	1
**IHD**	1
**NHP**	1
**HADS**	1
**OTHER**	9

**Table 3:** Average 36-Item Short Form Health Survey (SF-36) scores with 95% CI after craniotomy based upon tumor location. Skull base tumors were categorized into anterior, middle, and posterior cranial bases using Al-Mefty’s definition of skull base meningiomas.

Meningioma Location	Physical Functioning	Role Limitation Due to Physical Health	Role Limitation Due to Emotional Health	Energy	Social Function	Pain	General Health	Mental Health	PCS	MCS
Posterior	69 (95% CI 57–81)	53 (95% CI 35–71)	73 (95% CI 65–81)	52 (95% CI 40–64)	72 (95% CI 62–82)	72 (95% CI 70–75)	55 (95% CI 37–74)	65 (95% CI 60–70)	43 (95% CI 38–49)	47 (95% CI 43–52)
Anterior/Middle	84	79	83	68	82	75	79	68	49	52
NSB	77	65	75	59	76	72	64	72	46	49

**Table 4:** Recommendation of health-related quality-of-life instruments based on tumor location and treatment modality.

Treatment Modality	Anterior/Middle Skull Base	Posterior Skull Base
Craniotomy	ASBQ; SF-36	SF-36
EEA	ASBQ; SNOT-22; SF-36	SNOT-22; SF-36
Radiotherapy	ASBQ; QLQ BN-20; QLQ C30; SF-36	QLQ BN-20; QLQ C30; SF-36

Abbreviations: ASBQ = Anterior Skull Base Questionnaire; QLQ-C30 = EORTC Quality of Life Core questionnaire; QLQ-BN20 = EORTC Brain Cancer Module; SF-36 = Swedish version of the 36-item short; SNOT-22 = 22-item Sinonasal Outcome Test.

## Data Availability

Data from our systematic review and meta-analysis will be made available upon reasonable request.

## Abbreviations

HRQoL = Health-related quality of life; QOL = Quality of Life; Karnofsky Performance Scale = KPS; PROMS = Patient-reported outcome measure, SBM = Skull base meningioma; NSBM = Non-skull base meningiomas; SNOT-22 = 22-item sinonasal outcome test; SF-36 = Swedish version of the 36-item short form; EEA = Endoscopic endonasal approach; EOR = Extent of resection; ISPC = Iowa Rating Scales of Personality Change; APC = Acquired personality disturbance; vmPFC = ventromedial prefrontal cortex; GTR = Gross total resection; NTR = Near-total resection; STR = Sub-total resection; KPS = Karnofsky performance status; GKRS = Gamma knife radiosurgery; LPPM = Lateral Posterior Pyramide Meningioma LPPM; PCM = Petroclival Meningioma; EORTC = The European Organization for Research and Treatment of Cancer; QLQ-C30 = EORTC Quality of Life Core questionnaire; QLQ-BN20 = EORTC Brain Cancer Module; WHOQOL-100 = World Health Organization Quality of Life-100 Scale; IHD = Innsbruck Health Dimensions Questionnaire for Neurosurgical Patients; NHP = Nottingham Health Profile; ASBQ = Anterior Skull Base Questionnaire; IMRT = Intensity-modulated radiation therapy; Fractionated Stereotactic Radiosurgery = FMRT; Radiotherapy = RT.

## Appendix A

**Table A1:** ROBINS-I Risk of bias for all studies.

Author/Year	Confounding	Selection of Participants into the Study	Classification of Intervention	Deviations from Intended Intervention	Missing Outcome Data	Measurement of the Outcome	Selection of the Reported Result	Overall
Barrash et al., 2020 [30]	Serious	Moderate	Low	Low	Low	Low	Low	Serious
Batish et al., 2022 [20]	Moderate	Serious	Low	Low	Moderate	Low	Low	Serious
Chan et al., 1984 [44]	Moderate	Moderate	Low	Low	Moderate	Low	Low	Moderate
Combs et al., 2013 [60]	Low	Low	Low	Low	Low	Low	Low	Low
Fisher et al., 2022 [16]	Low	Low	Low	Low	Low	Low	Low	Low
Kirsbaum et al., 2021 [39]	Moderate	Low	Low	Low	Low	Low	Low	Moderate
Jesus et al., 1996 [31]	Serious	No Info	Low	Low	Low	Low	Low	Serious
Henzel et al., 2013 [46]	Low	Low	Low	Low	Moderate	Low	Low	Moderate
Jones et al., 2016 [38]	Moderate	Moderate	Low	Low	Moderate	Low	Low	Moderate
Kalasauskas et al., 2021 [18]	Moderate	Low	Low	Low	Moderate	Low	Low	Moderate
Karsy et al., 2019 [64]	Moderate	Moderate	Low	Low	Low	Low	Low	Moderate
Konglund et al., 2012 [32]	Low	Low	Low	Low	Moderate	Low	Low	Moderate
Krupp et al., 2008 [33]	Moderate	Moderate	Low	Low	Moderate	Low	Low	Moderate
Mathiesen et al., 2007 [19]	Serious	No Information	Low	Low	Low	Low	Low	Serious
Maio et al., 2009 [34]	Moderate	No Information	Low	Low	Low	Low	Low	Moderate
Mohsenipour et al., 2001 [35]	Moderate	No Info	Low	Low	Serious	Low	Low	Serious
Neil-Dwyer et al., 2000 [65]	Serious	Low	Low	Low	Low	Low	Low	Serious
Pintea et al., 2018 [12]	Moderate	Moderate	Low	Low	Moderate	Low	Low	Moderate
Tariciotti et al., 2022 [23]	Moderate	Moderate	Low	Low	Low	Low	Low	Moderate
Timmer et al., 2019 [36]	Serious	Moderate	Low	Low	Moderate	Low	Low	Serious
Wirsching et al., 2020 [37]	Moderate	Moderate	Low	Low	Moderate	Low	Low	Moderate
Zhao et al., 2020 [21]	Moderate	Moderate	Low	Low	Low	Low	Low	Moderate
Locatelli et al., 2020 [25]	Moderate	Moderate	Low	Low	Low	Low	Low	Moderate
Gao et al., 2021 [42]	Serious	Moderate	Low	Low	Low	Low	Low	Serious
Qiao et al., 2019 [66]	Low	Moderate	Low	Low	Low	Low	Low	Moderate
Koutourousiou et al., 2017 [26]	Moderate	Moderate	Low	Low	Low	Low	Low	Moderate
Li et al., 2016 [40]	Moderate	Moderate	Low	Low	Low	Low	Low	Moderate
Kalani et al., 2015 [24]	Serious	Moderate	Low	Low	Low	Low	Low	Serious

**Table A2:** Overview of 28 studies investigating health-related quality of life outcomes for meningioma patients following treatment.

Investigator	Number of Patients	WHO Grade	Average Nidus Size (mm)	Treatment Type	Intracranial Fossa Location	Extent of Resection	QOL Metric(s)	Median Follow-Up (mo)	Postoperative Deficits (Transient, Permanent, or Unspecified)	Average Time (mo) of QOL Metric Administered after Intervention
**Barrash et al., 2020** [30]	18	Grade I *n* = 17 Grade II *n* = 1	40.7	Craniotomy *n* = 18	Anterior *n* = 18	GTR = 17	ISPC; institution-specific	46.6	None	46.6 months
**Battish et al., 2022** [20]	32	NR	40	Craniotomy *n* = 29 Adjuvant GKRS *n* = 7	Posterior *n* = 32	GTR = 13 NTR = 17 STE *n* = 2	KPS; SF-36	34.7	All Unspecified IV deficit *n* = 3 VI deficit *n* = 4 VII deficit *n* = 4 IX-XI deficit *n* = 1 Motor weakness *n* = 5	34.7 months
**Chan et al., 1984** [44]	257	NR	70, *n* = 63 45–70, *n* = 71 <45 = *n* = 123	Craniotomy *n* = 257	Anterior *n* = 32 Middle *n* = 47 Posterior *n* = 41 NSB = 137	Grade I = 89 Grade II = 118 Grade III = 6 Grade IV = 43 Grade V = 1	KPS	108	Transient motor deficit *n* = 28 Transient dysphasia *n* = 6 Permanent motor deficit = 4 Seizure *n* = 1 Other *n* = 21	NR
**Combs et al., 2013** [60]	507	Grade I *n* = 234 Grade II *n* = 20 Grade III *n* = 15	NR	Craniotomy + FSRT or IMRT = 231 FST or IMRT after initial diagnosis = 145 Wait-and-see +FSRT or IMRT = 131	Anterior *n* = 51 Middle = 298 Posterior = 112	STR = 266	Institution-specific	107	NR	107 months
**Fisher et al., 2022** [16]	173	Grade I = 147 Grade II = 12	39	Craniotomy *n* = 140 Craniotomy + RT = 27 RT = 7	Anterior = 29 Middle = 33 Posterior = 27 NSB = 84	Simpson Grade I-III = 108 Simpson Grade IV-V= 40	SF-36; EORTC QLQ-BN20; Institution-specific	108	All Unspecified CN VII palsy = 2 CN III palsy = 2 Unilateral visual deficit = 3 Unilateral hearing deficit = 1 CN V palsy = 2 Sensory Deficit = 1 Motor Deficit = 1 Seizures = 2 Delirium = 1 Aphasia = 1 CN VI-CN X palsy = 3 Other = 6	108 months
**Castle-Kirszbau et al., 2022** [39]	50	NR	NR	Nasal endoscopic *n* = 50	Anterior = 50	GTR = 39 STR = 11	ASBQ; SNOT-22	12	Transient diabetes insipidus *n* = 3 Transient SIADH *n* = 6 Transient mild visual impairment *n* = 2	6 months
**Jesus et al., 1996** [31]	119	Grade I	NR	Craniotomy *n* = 119 Adjunct RT = 17	Middle = 119	GTR = 73 STR = 46	KPS	33.8	Transient pituitary dysfunction *n* = 17 Unspecified Infection *n* = 5	NR
**Henzel et al., 2013** [46]	52	Grade I = 33 Grade II = 7 Grade III = 2	NR	RT = 44	Anterior = 1 Middle = 29 Posterior= 17 NSB = 4	STR = 44	SF-36	24	Is aNR	6, 12, 18 and 24 months
**Jones et al., 2016** [38]	56	NR	25.4	Nasal endoscopic *n* = 34	Anterior = 29 Middle: *n* = 4 Posterior = 3	GTR = 15	ASBQ; SNOT-22	24	All Unspecified Anosmia *n* = 2 Worsening vision *n* = 2	42.3 months, at least 6 months
**Kalasauskas et al., 2020** [18]	62	Grade I *n* = 27 Grade II *n* = 4	24	Craniotomy *n* = 62	Anterior *n* = 6 Middle *n* = 22 Posterior *n* = 9 NSB *n* = 30	GTR = 31	DT; HADS; BFI; SF-36	NR	None	NR
**Karsy et al., 2019** [64]	52	Grade I *n* = 48 Grade II *n* = 4	46	Craniotomy *n* = 52	Anterior: 17 Middle: 16 Posterior: 13 NSB: 5	Simpson grade I–III = 28 Simpson Grade IV–V = 24	EQ-5D-3L	11.1	All Transient: New or worsening cranial nerve deficit *n* = 22 Seizures *n* = 1	1 and 12 months
**Konglund et al., 2013** [32]	54	Grade 1 = 51 Grade 2 = 2 Unknown = 1	NR	Craniotomy *n* = 54	Posterior = 5 NSB = 31 SB unspecified = 18	Simpsons grade I–III = 47 Simpsons grade IV = 7	KPS;QLQ-C30; HAD-A; HAD-D	NR	Transient neurological deterioration *n* = 10 Permanent neurological deterioration *n* = 7	6 months
**Krupp et al., 2009** [33]	91	Grade I = 91	NR	Craniotomy *n* = 91	Anterior = 12 Middle = 19 NSB = 60	NR	QLSS	13.4	Permanent hemiparesis *n*= 1	15 months
**Mathiesen, et al.,** **2007** [19]	29	NR	44.14	Craniotomy *n* = 29 Adjunct GKRS *n* = 7	Posterior = 29	Grade IV+ = 14 Grade II = 11 Grade IV = 1 Grade III = 1 Grade III+ = 1 Grade I =1	SF-36	66	Unspecified Hemiparesis *n* = 1 CN III dysfunction; Transient *n* = 7, Permanent *n* = 1 CN IV dysfunction Transient *n* = 6, Permanent *n* = 3 Transient CN VI dysfunction *n* = 6 CN V dysfunction Unspecified *n* = 7, Permanent *n* = 2 Transient Neuropathic Pain *n* = 2 CN VII dysfunction Transient *n* = 2, Permanent *n* = 6 Transient CN IX dysfunction *n* = 6	64.8 months
**Miao et al., 2010** [34]	147	NR	49	Craniotomy *n* = 147	Anterior = 13 Middle =19 Posterior = 14 NSB = 95	Simpson 0 = 12 Simpson I = 26 Simpson II = 30 Simpson III = 39 Simpson IV = 40	WHOQOL-100	NR	All unspecified *n* = unspecified Headache Visual disturbance Gait disturbance Cognitive function Loss of consciousness	NR
**Mohsenipour et al., 2001** [35]	82	NR	27.3	Craniotomy *n* = 82	Posterior = 13 NSB = 66	NR	IHD; NHP	NR	NR	NR
**Neil-Dwyer et al., 2000** [65]	19	NR	>30	Craniotomy *n* = 19 Adjuvant RT = 5	Posterior = 19	NR	SF-36	NR	Permanent neurological deficits *n* = 6 Transient neurological problems or exacerbation of existing deficits *n* = 10	12 months
**Pintea et al., 2018** [12]	78	NR	40	Craniotomy *n* = 66 RT = 12	Posterior = 78	Grade I–II = 47	SF-36	59	All Unspecified Eye Motility Impairment *n* = 23 Hyperacusis/Anacusis *n* = 6 CN VII Palsy *n* = 14 CN IX Dysfunction *n* = 12 Paresis/hemiparesis *n* = 12	59 months
**Tariciotti et al., 2022** [23]	165	Grade I = 126 Grade II = 31 Grade III = 2	NR	Craniotomy *n* = 165	NSB = 89 SB: Anterior = 30 Middle = 25 Posterior = 20	GTR = 128 STR = 37	KPS	33	NR	NR
**Timmer et al., 2019** [36]	133	Grade I = 109 Grade II = 22 Grade III = 2	NR	Craniotomy *n* = 133	NSB = 37 SB: Frontal *n* = 11 Middle: *n* = 22 Posterior = 22	Grade I = 37 Grade II =3 8 Grade III = 2 Grade IV = 10 NR = 46	SF-36; ASA; ADL	NR	NR	45.6 months
**Wirsching et al., 2020** [37]	249	Grade I *n* = 219 Grade II *n* = 30	NR	NR	NSB = 89 SB = 89: Posterior = 33	GTR = 189 STR = 49	EORTCQLQ-C30; MDASI-BT;EPICES	12	Unspecified *n* = 99	12 months
**Zhao et al., 2020** [21]	168	Grade I = 168	44.0	Craniotomy *n* = 152 GKRS = 4	Posterior = 168	GTR = 119 STR = 37	KPS	86.5	Unspecified Ataxia = 24 Unspecified Hemiparesis = 14 Unspecified CN III = 32 Unspecified CN IV = 38 Transient CN V = 11 Transient CN VI = 26 Transient CN VII = 34 Transient CN VIII = 20 Transient CN IX–XII = 6	86.5 months
**Locatelli et al., 2020** [25]	35	Grade I = 31 Grade II = 4	NR	Craniotomy *n* = 17 endoscopic superior eyelid *n* = 13 combined cranioendoscopic *n* = 5	anterior cranial fossa *n* = 18 middle cranial fossa *n* = 30	GTR = 20 STR = 15	KPS	Mean 31.5	Early Postop Deficits Intraoperative complications *n* = 1 Systemic complications *n* = 5 Surgical scar complications *n* = 1 Diplopia *n* = 10 ocular extrinsic muscle deficits *n* = 7 Visual-field deficits *n* = 6 Visual deficits *n* = 10 Other CN deficits *n* = 7 Intracranial complications *n* = 4 Long-Term Deficits Visual deficits *n* = 1 CSF leak *n* = 1 Hemisyndrome *n* = 1 Mild hypoesthesia in V2 *n* = 2	Mean 31.5
**Gao et al., 2021** [42]	107	Grade I *n* = 95 Grade II *n* = 9 Grade III *n* = 3	39.1 mm	Craniotomy = 107	Posterior cranial fossa *n* = 107	Total *n* = 57 Subtotal *n* = 39 Partial *n* = 11	KPS	61 Months	CN dysfunction *n* = 29 intracranial infection *n* = 14 CSF leakage *n* = 9 hematoma *n* = 3	NR
**Qiao et al., 2019** [66]	176	Grade I *n* = 156 Grade II *n* = 18 WHO grade III *n* = 2	NR	Surgery *n* = 127 surgery + RT *n* = 15 surgery + GKS *n* = 34.	Posterior cranial fossa *n* = 176	GTR *n* = 61 STR *n* = 102 PR *n* = 13	KPS	NR	All Unspecified Hydrocephalus *n* = 64 Intracranial Infection *n* = 2 Tonsillar Herniation *n* = 15 Conjunctivitis or Keratitis or Corneal ulcer *n* = 3 intracranial hematoma; *n* = 1 Gastric ulcer *n* = 1 DVT of extremity *n* = 1 Pneumonia *n* = 8 subdural or subcutaneous *n* = 2	36
**Koutourousiou et al., 2017** [26]	32	Grade I *n* = 29 Grade II *n* = 2 grade III *n* = 1	41.7 mm	Craniotomy = 11 Endoscopic = 17 Craniotomy + Endoscopic = 4	Posterior cranial fossa *n* = 32	GTR *n* = 6 Near total *n* = 9 STR *n* = 8 Partial *n* = 9	KPS	14 Months	All Unspecified CN III *n* = 1 CN IV *n* = 1 CN V *n* = 3 CN VI *n* = 14 CN VII *n* = 1 CN VIII *n* = 1 CN IX, X *n* = 1 CSF Leak *n* = 9 Hydrocephalus *n* = 5 Meningitis *n* = 3 PE/DVT *n* = 3 Perioperative Death *n* = 1	1 month
**Li et al.,** **2016** [40]	199	Grade I *n* = 199	47 mm	NR	Posterior cranial fossa *n* = 199	GTR *n* = 111 STR *n* = 65 Partial *n* = 23	KPS SF-36	171.6	All Permanent CN III *n* = 11 CN IV *n* = 9 CN VI *n* = 10 Oculomotor deficit *n* = 15 CN V Facial numbness *n* = 4 Weak corneal reflex *n* = 6 CN VII *n* = 5 CN VIII *n* = 2 CN IX–XII *n* = 1 Hemiparesis *n* = 7 Ataxia *n* = 8	1 month
**Kalani et al., 2015** [24]	25	Grade 1 *n* = 25	NR	Craniotomy = 25	Anterior cranial fossa *n* = 3 Middle cranial fossa *n* = 5 Posterior cranial fossa *n* = 4	Simpson grade; Grade I *n* = 4 Grade II *n* = 16 Grade III *n* = 3 Grade IV *n* = 2	KPS	Mean = 101.7 months	visual disturbances such as diplopia, visual decline, and cranial nerve palsies remained more refractory to surgical treatment.	NR
